# Management of Postoperative Pancreatic Fistulas After Pancreaticoduodenectomy Using Open Drainage and Negative Pressure Wound Therapy With Instillation and Dwell Times

**DOI:** 10.7759/cureus.67135

**Published:** 2024-08-18

**Authors:** Yoshihiro Miyasaka, Hiroki Kaida, Makoto Kawamoto, Masato Watanabe

**Affiliations:** 1 Department of Surgery, Fukuoka University Chikushi Hospital, Chikushino, JPN; 2 Department of Surgery and Oncology, Graduate School of Medical Sciences, Kyushu University, Fukuoka, JPN

**Keywords:** npwti-d, negative pressure wound therapy with instillation and dwell time, open drainage, pancreatoduodenectomy, popf, postoperative pancreatic fistula

## Abstract

Introduction

Postoperative pancreatic fistula (POPF) is a common complication of pancreatoduodenectomy (PD) that may cause lethal complications. Therefore, it is important to properly treat POPF and prevent its aggravation during the postoperative management of PD. We have used a combination of open drainage, in which the wound above the fluid collection is opened, and negative pressure wound therapy with instillation and dwell time (NPWTi-d) to manage POPF after PD. To evaluate the feasibility and efficacy of this combination treatment, we analyzed the outcomes of patients with POPF after PD.

Methods

Patients who underwent PD were reviewed and those who developed POPF were extracted and divided into three groups according to the management of POPF: N group (patients treated with open drainage and NPWTi-d), O group (patients treated with open drainage without NPWTi-d), and C group (patients treated with catheter drainage). The perioperative outcomes were compared among the three groups.

Results

During the study period, 133 patients underwent PD, out of which 39 (29%) developed POPF (≥grade B). Among the 39 patients with POPF, eight, four, and 27 were classified into the N, O, and C group, respectively. No mortality was observed in the patients with POPF. No severe complications were observed in the patients who underwent open drainage (N and O groups), while two patients in the C group developed severe complications. Among the patients who underwent open drainage, the N group tended to have a shorter postoperative hospital stay than the O group.

Conclusions

The current study suggests that open drainage safely and effectively healed POPF and NPWTi-d promoted wound closure. The combination of open drainage and NPWTi-d may prevent the aggravation of POPF, reduce failure to rescue, and shorten hospital stay after PD.

## Introduction

Pancreaticoduodenectomy (PD) is a surgical procedure performed to treat benign and malignant diseases of the pancreatic head, distal bile duct, and duodenum. It is not only technically demanding, but also associated with a variety of postoperative complications. Among such complications, postoperative pancreatic fistula (POPF) is frequently recognized after PD and may lead to lethal complications, such as sepsis or rupture of the pseudoaneurysm. The incidence of clinically relevant POPF after PD is reported to range from 10% to 34%, and several factors such as male sex, obesity, small pancreatic duct, and soft pancreas have been reported as risk factors for POPF [1]. Although various attempts have been made to prevent POPF, effective preventive measures have yet to be established. Therefore, it is important to properly treat POPF and prevent its aggravation during postoperative management of PD. The management of POPF varies widely. Drainage is required if an infectious fluid collection/abscess is present. Although catheter drainage is commonly used for POPF, it is sometimes insufficient and may lead to death. Open drainage, in which the wound above the fluid collection is opened to ensure drainage, is reported as a useful method for managing POPF, and our institution has employed this method since 2019 [2].

Negative pressure wound therapy with instillation and dwell time (NPWTi-d) is used to manage complex wounds, including infected wounds and has been reported to be useful for wounds of various etiologies [3,4]. It is a combination of negative pressure, which removes excessive fluid, improves tissue blood circulation, and encourages granulation tissue formation, and instillation of a topical solution, which helps wound cleansing and reduces the bacterial burden; therefore, NPWTi-d is expected to promote wound healing. To promote wound healing, we applied NPWTi-d on a drainage wound after open drainage for POPF.

This study aimed to analyze the outcomes of POPF management, including the combination of open drainage and NPWTi-d, to evaluate the feasibility and efficacy of the method employed.

## Materials and methods

Study population

Patients who underwent PD at Fukuoka University Chikushi Hospital between January 2008 and December 2023 were included. Patient characteristics, operative information, and postoperative clinical courses were retrieved from electronic medical records. Patients who developed POPF were extracted and divided into three groups according to how POPF was managed: N group (patients treated with open drainage and NPWTi-d), O group (patients treated with open drainage without NPWTi-d), and C group (patients treated with catheter drainage, including persistent drainage using an intraoperatively placed drain, drain exchange, or percutaneous drainage). The perioperative outcomes were compared among the three groups. The presence of POPF was determined according to the International Study Group of Pancreatic Fistula (ISGPF) definition, and POPF of ISGPF grade B or C was recorded as POPF [5]. Postoperative complications were graded according to the Clavien-Dindo classification [6].

Surgical procedure

During the study period, an open approach was adopted for all the patients with PD. The conventional Whipple procedure was performed until March 2019, after which pylorus-preserving PD (PpPD) was performed. Pylorus-resecting (subtotal stomach-preserving) PD (PrPD) was performed in some patients. Reconstruction was performed according to the modified Child’s method. Several methods of pancreatojejunostomy (e.g., the Blumgart method) were selected according to the surgeon’s preference. External pancreatic drainage was performed in the early period, and a lost stent was used in the latter period. Although drains were placed around the pancreatojejunal anastomosis in all patients, the number and type of drains varied among the surgeons.

Open drainage for POPF

**Figure 1:**
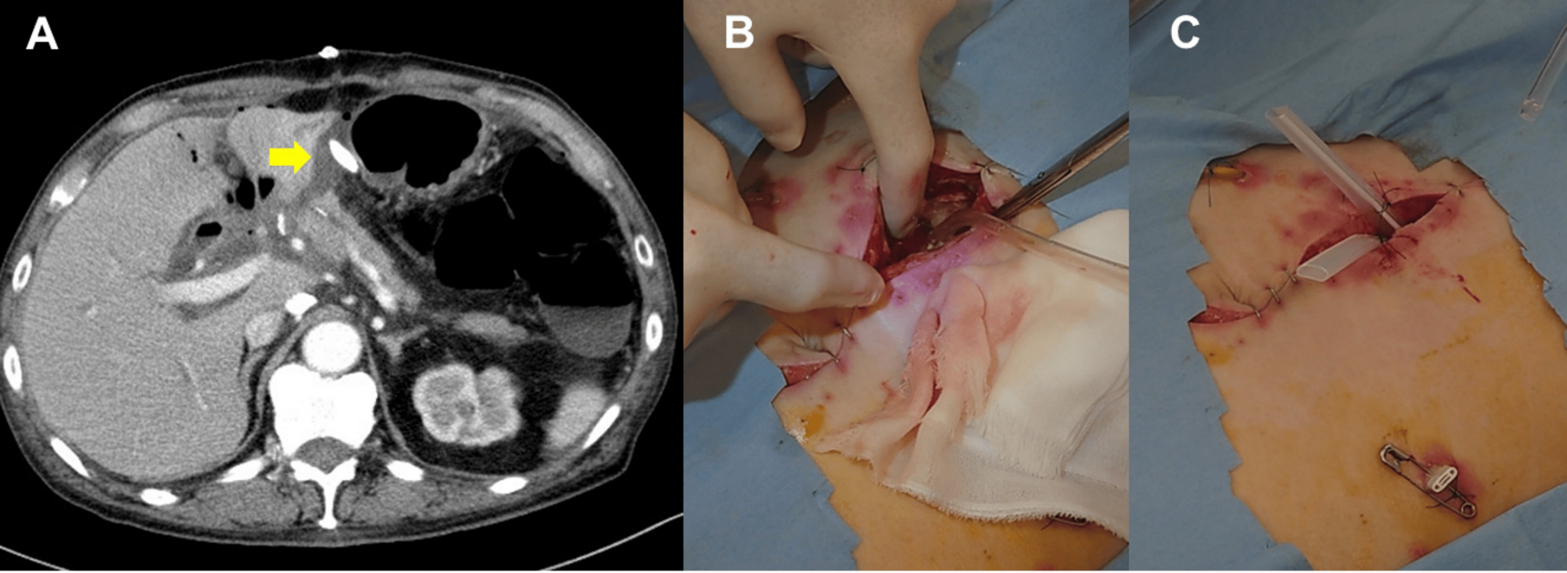
The open drainage procedure. A. A contrast-enhanced computed tomography image shows fluid collection between the lateral section of the liver and pancreatojejunostomy (arrow) despite a catheter drain, which was intraoperatively placed. B. After removing the fascial sutures, the fluid collection is opened manually under direct vision. C. A view after open drainage. A Penrose drain is placed to maintain the drainage route. The drain at the left side of the abdomen will be exchanged under fluoroscopy later.

Open drainage is performed when fluid collection is observed in the space between the left lateral section of the liver and pancreatojejunostomy, which is just below the median incision (Figure 1A). The open drainage procedure has been described previously [2]. The patient is administered intravenous pentazocine and local anesthesia. To create a sufficient drainage route, the surgical wound is opened by approximately 5 cm, and the fascia sutures are removed. Blunt dissection is performed manually to reach and open the fluid collection site (Figure 1B). After opening the collection site, drains are placed to maintain the drainage route (Figure 1C). The open wound is covered with a wet gauze dressing and checked twice daily.

NPWTi-d

**Figure 2:**
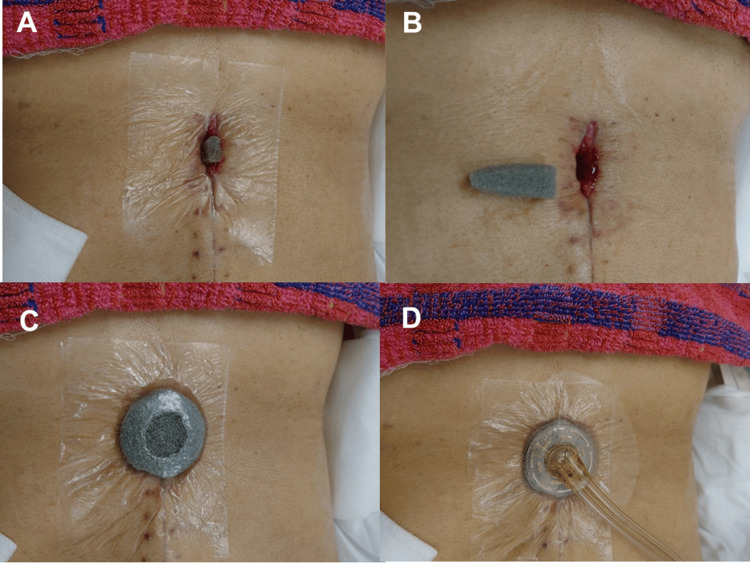
Procedures of NPWTi-d. A. Covering the skin around the wound with polyurethane film. B. Trimming the polyurethane foam to fit the wound space. C. A circular foam is placed on the wound and covered with film. A 3-cm hole is created. D. A pad of the NPWTi-d system is attached. NPWTi-d: negative pressure wound therapy with instillation and dwell time

After the inflammation subsides and fluoroscopic examination confirms no communication between the wound and the gastrointestinal tract, NPWTi-d is applied. The skin around the open drainage wound is covered with polyurethane film (V.A.C.® Advanced Drape; KCI, San Antonio, TX, USA) to avoid skin damage due to negative pressure (Figure 2A). Polyurethane foam (V.A.C. Veraflo^TM^ Dressing; KCI, San Antonio, TX, USA) trimmed to fit the shape of the wound space is inserted into the wound, and a circular foam approximately 6 cm in diameter is placed on it (Figure 2B and C). If the intestinal tract is close to the wound space, non-adhering dressing (SI Aid-Mesh^TM^, ALCARE, Tokyo, Japan) is placed on the abdominal contents to avoid damage to the intestines and formation of intestinal fistula. The circular foam is covered with film, and a 3 cm hole is created in the film at the center of the foam. A pad with a double-lumen tube (V.A.C. VeraT.R.A.C.^TM^ Pad, KCI, San Antonio, TX, USA) is attached (Figure 2D). The pad is connected to an automated NPWTi-d system (V.A.C. ULTA^TM^ Therapy System, KCI, San Antonio, TX, USA). After the instillation of normal saline, a 10-minute dwelling time is set, followed by 110 minutes of -125 mmHg continuous negative pressure. The dressings are changed twice weekly. NPWTi-d is terminated when the wound has shrunk and is covered with granulation tissue.

Statistical analysis

Continuous data were expressed as median (range) and compared using the Mann-Whitney U test. Categorical data were expressed as absolute values and percentages and were compared using Fisher’s exact probability test. Statistical significance was set at P < 0.05. All the analyses were conducted using the JMP statistical software (version 14.0; SAS Institute, Cary, NC, USA).

## Results

**Figure 3:**
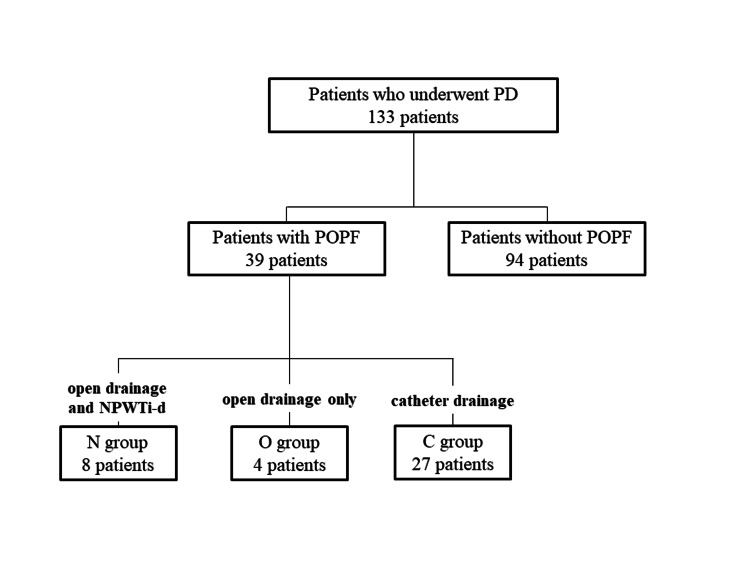
Flow diagram of the study.

During the study period, 133 patients underwent PD, among which 39 (29%) developed POPF (≥grade B) (Figure 3). The characteristics and perioperative outcomes of the patients who developed POPF and those who did not are shown in Table 1. Patients who developed POPF had a significantly higher body mass index and a relatively higher proportion of men. The length of postoperative hospital stay was significantly longer in patients with POPF. In-hospital mortality was observed in one patient who had gastrointestinal bleeding, while no patient with POPF died during the postoperative hospital stay.

**Table 1:** Patient characteristics and perioperative outcomes *Comparison between POPF (+) and POPF (-). Ranges are given in parentheses. IPMN: intraductal papillary mucinous neoplasm, PD: pancreaticoduodenectomy, PpPD: pylorus-preserving pancreaticoduodenectomy, PrPD: pylorus-resecting pancreaticoduodenectomy.

	Total	POPF (+) (n=39)	POPF (-) (n=94)	p value*
Age (years)	70 (51-84)	67 (55-81)	70 (51-84)	0.1431
Sex (female)	66 (50%)	14 (36%)	52 (55%)	0.0668
Body mass index (kg/m^2^)	20.8 (14.6-34.9)	22.4 (14.6-34.9)	20.4 (14.6-28.8)	0.0002
Diagnosis				0.1083
Pancreatic cancer	57	10	47	
Bile duct cancer	25	11	14	
Ampullary cancer	23	9	14	
Duodenal cancer	4	2	2	
IPMN	11	3	8	
Ampullary adenoma	6	3	3	
Others	7	1	6	
PD procedure				0.2543
Classic PD	76	19	57	
PpPD	54	18	36	
PrPD	3	2	1	
Operation time (min)	434 (195-1126)	434 (235-1126)	433 (195-762)	0.937
Blood loss (ml)	785 (50-3780)	785 (50-3780)	770 (50-3002)	0.9941
Postoperative hospital stay (days)	33 (15-347)	42 (29-347)	29 (15-113)	<0.0001

Of the patients with POPF, eight patients underwent open drainage and NPWTi-d (N group), four patients had open drainage but not NPWTi-d (O group), and 27 were treated with catheter drainage only (C group) (Figure 3). A comparison of the characteristics and perioperative outcomes among the three groups is shown in Table 2. There were no significant differences in patient characteristics, except for the PD procedure. Due to changes in surgical procedures over time, all patients in the N group had PpPD, whereas the majority of patients in the C group had classic PD. Although no perioperative mortality was recognized, two patients in the C group had severe complications (Clavien-Dindo classification ≥ grade Ⅲb): one patient required intensive care unit admission due to sepsis, and the other required reoperation for rupture of pseudoanuerysm. In contrast, no severe complications were observed in patients in either the N or O group. The O group had a relatively longer postoperative hospital stay than the other groups.

**Table 2:** Comparison of patient characteristics and perioperative outcomes according to the management of POPF Ranges are given in parentheses. IPMN: intraductal papillary mucinous neoplasm, PD: pancreaticoduodenectomy, PpPD: pylorus-preserving pancreaticoduodenectomy, PrPD: pylorus-resecting pancreaticoduodenectomy.

	N group (n=8)	O group (n=4)	C group (n=27)	p value
Age (years)	72 (57-80)	68 (59-75)	65 (55-81)	0.2969
Sex, female	1 (13%)	2 (50%)	11 (41%)	0.2407
Body mass index (kg/m^2^)	22.0 (20.1-26.6)	23.2 (19.5-24.1)	22.4 (14.6-34.9)	0.9692
Diagnosis				0.5425
Pancreatic cancer	3	2	5	
Bile duct cancer	3	2	6	
Ampullary cancer	1	0	8	
Duodenal cancer	0	0	2	
IPMN	0	0	3	
Ampullary adenoma	1	0	2	
Others	0	0	1	
PD procedure				0.0016
Classic PD	0	2	18	
PpPD	8	2	7	
PrPD	0	0	2	
Operation time (min)	442.4 (371-819)	464 (367-550)	401 (235-1126)	0.3544
Blood loss (ml)	516 (50-842)	385 (215-1300)	869 (63-3780)	0.0665
Clavien-Dindo classification ≥ grade Ⅲb	0 (0%)	0 (0%)	2 (7%)	0.4680
Postoperative hospital stay (days)	42 (32-92)	49.5 (31-67)	40 (29-347)	0.6070

Table 3 shows the characteristics of the patients in the N group. Three patients developed complications other than POPF. Concomitant catheter drainage was used in five patients (persistent drainage using intraoperatively placed drains that were exchanged later in four patients and ultrasonography-guided percutaneous drainage in one patient). The median durations from operation to open drainage, from open drainage to start of NPWTi-d and of NPWTi-d were 6.5 days (3-8), 8 days (7-24), and 19.5 days (14-25), respectively. One patient showed a long postoperative hospital stay despite comparable durations of open drainage and NPWTi-d to the others because anastomotic leakage of the duodenojejunostomy followed by stenosis developed and took time to be restored (Case 6).

**Table 3:** Characteristics of patients who underwent open drainage and NPWTi-d NPWTi-d: negative pressure wound therapy with instillation and dwell time.

Case	Age (years)	Sex	Diagnosis	Other complication	Concomitant catheter drainage	Operation to open drainage (days)	Open drainage to NPWTi-d (days)	Duration of NPWTi-d (days)	Postoperative hospital stay (days)
1	79	M	Pancreatic cancer	None	Yes	8	7	25	44
2	57	M	Pancreatic cancer	None	Yes	3	18	16	40
3	67	M	Ampullary Adenoma	None	No	8	10	14	40
4	80	M	Bile duct cancer	None	Yes	4	7	21	38
5	73	M	Pancreatic cancer	None	No	6	9	23	44
6	67	M	Bile duct cancer	Anastomotic leakage and stenosis	Yes	7	7	24	92
7	77	M	Ampullary Adenoma	Enterocolitis infectious	No	7	7	14	32
8	71	F	Bile duct cancer	Catheter-related infection	Yes	4	24	18	46

## Discussion

Here, we report our preliminary experience of managing POPF using a combination of open drainage and NPWTi-d. In the current analysis, patients who underwent open drainage did not develop severe complications (≥ Clavien-Dindo classification grade Ⅲb). In addition, the median postoperative hospital stay of patients treated with NPWTi-d was one week shorter than that of patients treated with open drainage only, although the difference was not statistically significant. These findings suggest that the combination of open drainage and NPWTi-d may prevent the exacerbation of POPF and shorten the treatment period.

The purpose of the management of POPF is to stabilize the patient's condition as well as to avoid serious complications and failure to rescue and heal fistula. The modalities for the management of POPF are wide-ranging and include nutritional support (enteral and total parenteral nutrition), pharmacotherapy (antibiotics and somatostatin analogs), drainage, and reoperation [7]. Drainage is often employed in the management of POPF to expel the collected digestive juice from the abdominal cavity to control infection and avoid erosion of the surrounding organs, including vessels. The drainage methods for POPF include several procedures such as maintenance or optimization of intraoperatively placed drains, percutaneous drainage, endoscopic transluminal drainage, and drain placement during reoperation. Although a catheter is generally utilized for drainage, it may be occluded by pus of high viscosity, leading to insufficient drainage. In addition, they may pose a risk of injury to organs or vessels. Smits et al. reported that the success rate of primary catheter drainage for severe POPF (POPF requiring drainage or relaparotomy) was 77.1% [8]. The open drainage method described in this manuscript was previously reported by Kyushu University [2]. Although it seems relaparotomy and drain placement under general anesthesia, it can be performed under local anesthesia in a surgical ward. Moreover, it does not require an additional incision but only the removal of sutures from the existing wound. The invasiveness of this procedure is similar to that of percutaneous catheter drainage. In addition, it provides a wide drainage route through which we can safely perform debridement under direct vision, thereby avoiding injury to the organs and vessels. In our series, no subsequent severe complication occurred after open drainage, whereas two of the 27 patients treated with catheter drainage had subsequent complications of Clavien-Dindo classification ≥ grade Ⅲb. We believe that open drainage is a safe and effective procedure for fluid collection during POPF.

The weakness of this method is that it cannot be performed when the fluid collection is far from the median incision. However, pancreatojejunostomy, which is usually the origin of the pancreatic fistula after PD, is located in the vicinity of the median incision in most cases. Moreover, this approach is not applicable in cases without a median incision, such as in minimally invasive PD with intracorporeal reconstruction.

Although delayed wound healing due to infectious discharge is a concern regarding wound drainage, NPWTi-d provides a solution. NPWTi-d controls wound infection according to the instillation and dwelling of a solution, which effectively cleanses the wound and washes away bacteria and necrotic tissue, and continuous negative pressure, which removes contaminants from the wound and prevents the spread of infection [9]. In the case of POPF, these functions protect the wound and surrounding skin from pancreatic juice, which irritates the wound and skin. Furthermore, continuous negative pressure enhances granulation tissue formation by improving microcirculation around the wound and stimulating fibroblast proliferation [9,10]. Various reports have described the efficacy of NPWTi-d in the management of complex infectious wounds, including open abdominal wounds for abdominal sepsis [3,11]. Intestinal fistula formation is a concern if NPWTi-d is applied to an open abdominal wound. Therefore, we use non-adhering dressing when intestinal tracts are close to the wound space to prevent damage to the intestine. Thus, we did not experience intestinal fistula formation in patients treated with open drainage and NPWTi-d. The current study showed that NPWTi-d followed by open drainage shortened the postoperative hospital stay by one week compared to open drainage only, although the difference was not clinically significant (probably due to the small sample size). This finding indicates that NPWTi-d promotes the closure of open drainage wounds.

The limitations of this study are as follows. First, it was a single-institution retrospective study with a small cohort. Second, a combination of open drainage and NPWTi-d was performed only in recent patients. Therefore, outcomes might be influenced by factors other than drainage, such as changes in the management policies for POPF.

## Conclusions

Here, we describe our preliminary experience with the combination therapy of open drainage and NPWTi-d for POPF after PD. Our results suggest that open drainage safely and effectively healed POPF, and NPWTi-d promoted wound closure. The combination of open drainage and NPWTi-d may prevent the aggravation of POPF, reduce failure to rescue, and shorten hospital stay after PD.
